# Usefulness of Gamma‐Glutamyl Transferase and Sequential Carbohydrate‐Deficient Transferrin for Unhealthy Alcohol Use Screening in Indigenous Communities

**DOI:** 10.1002/kjm2.70119

**Published:** 2025-10-15

**Authors:** Jing‐Houng Wang, Hui‐Min Tien, Hsiao‐Chu Lien, Pei‐Lin Chou, Yu‐Ling Chen, Nien‐Tzu Hsu, Sheng‐Nan Lu

**Affiliations:** ^1^ Division of Hepato‐Gastroenterology, Department of Internal Medicine Kaohsiung Chang Gung Memorial Hospital and Chang Gung University College of Medicine Kaohsiung Taiwan; ^2^ Kaohsiung Research Association for the Control of the Liver Diseases Kaohsiung Taiwan; ^3^ Laiyi Primary Health Center Pingtung Taiwan; ^4^ Mudan Primary Health Center Pingtung Taiwan

**Keywords:** community screening, gamma‐glutamyl transferase (GGT), percentage of carbohydrate‐deficient transferrin (%CDT), unhealthy alcohol use (UAU)

## Abstract

Unhealthy alcohol use (UAU) screening facilitates identification of patients with alcohol use problems for intervention. This study evaluates the usefulness of gamma‐glutamyl transferase (GGT) and sequential percentage of carbohydrate‐deficient transferrin (%CDT) in community screening for UAU. In two indigenous communities (A and B), liver disease screening data including GGT, aspartate aminotransferase (AST), alanine aminotransferase (ALT), and alcohol use disorders identification test (AUDIT) were reviewed. In Community B, residents with high GGT were invited for sequential evaluation including %CDT, AUDIT, ultrasonography, and liver stiffness measurement. With AUDIT score ≥ 8 and ≥ 4 as UAU criteria for male and female genders, we determined the diagnostic performances and validities of GGT and sequential %CDT in detecting UAU. A total of 1755 residents with available GGT, AST, ALT, and AUDIT were reviewed. UAU prevalence ranged from 7.4% to 44% between communities. For sequential evaluation, 79 residents were enrolled in which 57 (72.2%) were classified as UAU. The performances of GGT, AST, ALT, and AST/ALT ratios in detecting UAU were 0.767, 0.66, 0.597, and 0.549, respectively. With a cutoff of GGT ≥ upper normal limit in detecting UAU, the sensitivity, specificity, accuracy, positive (PPV), and negative predictive value (NPV) were 62.5%, 75.5%, 72.1%, 47.4%, and 85.0%. In Community B, among residents with high GGT, %CDT had a performance of 0.684. With a cutoff of 1.72, the sensitivity, specificity, accuracy, PPV, and NPV were 59.6%, 81.8%, 65.8%, 89.5%, and 43.9%, respectively. GGT and sequential %CDT are useful for screening UAU in indigenous communities with high negative and positive predictive values, respectively.

**Abbreviations**
%CDT: percentage of carbohydrate‐deficient transferrin
ALT: alanine aminotransferase
AST: aspartate aminotransferase
AUDIT: alcohol use disorders identification test
AUROC: area under the ROC curve
CI: confidence interval
GGT: gamma‐glutamyl transferase
IQR: interquadrant range
NPV: negative predictive value
PPV: positive predictive value
ROC: receiver operating characteristic (ROC) curve
SD: standard deviation
UAU: unhealthy alcohol use
UNL: upper normal limit

## 1 Introduction

According to the World Health Organization 2014 report, harmful alcohol use causes about 3.3 million deaths every year and 139 million disability‐adjusted life years, corresponding to 5.9% of all deaths and 5.1% of the global burden of disease and injury [1]. Alcohol has an impact on over 200 diseases, with the largest number of deaths from cardiovascular disease, injuries, gastrointestinal disease, and cancers; however, the alcohol‐attributable fraction is highest for liver disease, especially cirrhosis [2, 3]. For indigenous populations, alcohol‐related diseases are the important factor for lower health standards [4, 5]. To facilitate early detection of those patients with alcohol use problems who could benefit from a brief intervention and further assessment/treatment, the public health approach begins with screening for unhealthy alcohol use (UAU) [6, 7] that can be conducted in the settings of general practice and community health service centers, hospital outpatient clinics and wards, as well as in emergency departments [7].

Alcohol use disorders identification test (AUDIT) questionnaire as a structured and validated questionnaire with good sensitivity and specificity is widely recommended as the standard screening tool to identify such disorders [2, 7]. Alcohol biomarkers in blood, urine, or hair might aid in diagnosis, while the blood gamma‐glutamyl transferase (GGT) test as a marker of the oxidative stress associated with ethanol metabolism that is elevated in regular drinkers with a long drinking history [8] is widely available and inexpensive. At varying cutoffs, GGT has sensitivity and specificity of 37%–95% and 18%–93% as an alcohol use biomarker [6]. Carbohydrate‐deficient transferrin (CDT) is generated as a result of alcohol inhibition of transferrin glycosylation and is a biomarker for long‐term alcohol consumption with sensitivity and specificity of 46%–90% and 70%–100% [6, 8], although while the percentage of CDT (%CDT) has higher diagnostic performance than GGT in clinical practice, combining these might increase the sensitivity to screen excess alcohol use in the general population [9, 10]. However, the sensitivity and specificity of GGT and CDT were affected by comorbid medical conditions not related to alcohol, chronic liver disease, medications causing enzymatic induction of GGT, and rare genetic variations of transferrin [8]. In addition, the positive and negative predictive values in screening depended on the UAU prevalence in the study community. In indigenous communities where alcohol‐related disease might be high and is an issue for health disparity, it is important to investigate the utility of GGT and %CDT for screening of the UAU.

In indigenous communities, the purposes of this study were to determine the usefulness of GGT and sequential %CDT for high GGT in screening for UAU with AUDIT as the reference standard, and to investigate liver injury related to high GGT.

## 2 Materials and Methods

### 2.1 Patients

The study protocol was approved by the Institutional Review Board of this hospital and Taiwan Council of Indigenous Peoples (CRB‐112‐006). Between 2020 and 2021, one liver disease screening program with blood tests including aminotransferase and GGT was conducted in two indigenous areas (Communities A and B) in southern Taiwan [11]. In this screening program, AUDIT was also performed to identify alcohol use disorders in those participating in the program. We reviewed liver disease screening data including GGT, aspartate aminotransferase (AST), alanine aminotransferase (ALT), and AUDIT. With AUDIT score ≥ 8 and ≥ 4 as the criteria of UAU for males and females, respectively, the performances of GGT and aminotransferase in identifying UAU were analyzed; then the cutoffs and diagnostic validities in the identification of UAU were determined.

In Community B, residents with high GGT levels (defined as GGT ≥ upper normal limit) within 6 months before the study were invited to attend a sequential exam in a liver clinic at a public health center. All residents signed informed consent before enrollment, where baseline demographics were recorded. AUDIT was performed to identify alcohol use disorders. Physical measurements and blood tests including complete blood count, prothrombin time, liver function tests, lipid profile, glycated hemoglobin, alpha‐fetoprotein, and %CDT (N Latex CDT assay, Siemens Medical Solutions USA), transient elastography (Echosens, Paris, France), and ultrasonography were performed, with %CDT in the identification of UAU with AUDIT scores ≥ 8 and ≥ 4 being established as the criteria of UAU for male and female genders. The cutoffs and diagnostic validities in the detection of UAU were then determined, and for those with UAU, brief interventions were performed including health education and referral for treatments.

### 2.2 Statistics

Quantitative variables were expressed with mean ± standard deviation (SD) or median with an interquartile range (IQR), while qualitative variables were expressed as absolute and relative frequencies. Mann–Whitney *U* test was used to compare continuous data, while *χ* ^2^ or Fisher's exact tests were used to compare categorical variables. The performance of markers in the identification of UAU was assessed with receiver operating characteristic (ROC) curve where the area under the ROC curve (AUROC) and the 95% confidence interval (CI) were used as indexes of accuracy. Optimal cutoffs and their diagnostic validities were determined, with all data being recorded and analyzed using the SPSS v10 software package (SPSS Inc., Chicago, IL, USA).

## 3 Results

### 3.1 Patients

In the two indigenous communities, a total of 1755 residents including 792 males and 963 females with available GGT, AST, ALT, and ADUIT were reviewed and enrolled. Table 1 shows demographics and clinical characteristics of enrolled residents. The median AST and ALT levels were 24 IU/L and 21 IU/L; 620 (35.3%) had high GGT; and the prevalence of UAU was 26.2%, 26.3%, and 26.1% in all, males, and females, respectively. Compared with Community B, there was a higher prevalence of GGT and UAU in Community A. While UAU prevalence ranged from 7.4% to 44% between communities, there were 4.6% (81/1755) residents with ADUIT ≥ 20 defined as moderate or severe alcohol use disorder (Supporting Information).

**TABLE 1:** The demographics, screen results, and unhealthy alcohol use (UAU) of screening attendees of two indigenous communities (A and B).

	Total (*N* = 1755)	Community A (*N* = 901)	Community B (*N* = 854)
Age (years, median, IQR)	57.0 (45.7–66.7)	60.8 (49.4–69.4)	54.5 (42.8–62.7)
Sex (%)			
Male	792 (45.1)	382 (42.4)	410 (48.0)
Female	963 (54.9)	519 (57.6)	444 (52.0)
AST (U/L, median, IQR)	24 (20–33)	25 (21–34)	24 (19–32)
ALT (U/L, median, IQR)	21 (15–32)	22 (15–31)	21 (15–33)
GGT (%)			
< 1 × UNL	1135 (64.7)	494 (54.8)	641 (75.1)
≥ 1 × UNL	620 (35.3)	407 (45.2)	213 (24.9)
GGT (%)			
< 2 × UNL	1463 (83.4)	699 (77.6)	764 (89.5)
≥ 2 × UNL	292 (16.6)	202 (22.4)	90 (10.5)
GGT (%)			
< 3 × UNL	1575 (89.7)	778 (86.3)	797 (93.3)
≥ 3 × UNL	180 (10.3)	123 (13.7)	57 (6.7)
UAU (%)	459 (26.2)	396 (44.0)	63 (7.4)
Male	208 (26.3)	176 (46.1)	32 (7.8)
Female	251 (26.1)	220 (42.4)	31 (7.0)

Abbreviations: ALT, alanine aminotransferase; AST, aspartate aminotransferase; GGT, gamma‐glutamyl transferase; IQR, interquartile range; UNL, upper normal limit.

At Community B, 133 residents with high GGT were invited to undergo the sequential exams. Seventy‐nine residents (59.4%), including 49 males and 30 females, were enrolled in the prospective cohort of the sequential exam. Median AST, ALT, and %CDT levels were 32 IU/L, 26 IU/L, and 1.67, with median liver stiffness being 6.3 kPa. Fifty‐seven (72.2%) residents had UAU, and compared to those without UAU, these had significantly higher %CDT, lower glycated hemoglobin, and low density lipoprotein levels (Table 2). Among these 57, 21 had UAU only, 23 had UAU combined with metabolic syndrome, four were in combination with viral hepatitis, and nine in combination with both metabolic syndrome and viral hepatitis. For non‐UAU residents, seven residents had metabolic syndrome only, three had viral hepatitis only, and 12 residents had other conditions.

**TABLE 2:** The demographics and clinical characteristics of residents in the sequential exam for high gamma‐glutamyl transferase (GGT) in Community B.

Variables	Total (*N* = 79)	UAU (*N* = 57)	Non‐UAU (*N* = 22)	*p*
Age (years, median, IQR)	61.5 (55.4–68.9)	60.9 (54.1–66.5)	64.3 (58.9–74.7)	0.079
Sex (%)				0.395
Male	49 (62.0)	37 (64.9)	12 (54.5)	
Female	30 (38.0)	20 (35.1)	10 (45.5)	
BMI (kg/m^2^, median, IQR)	27.8 (25.0–30.2)	27.7 (25.0–30.4)	27.9 (25.9–30.0)	0.739
Waist (cm, median, IQR)	94 (90–103)	94 (90–105)	94.5 (90–100)	0.978
MS (%)				0.053
No	40 (50.6)	25 (43.9)	15 (68.2)	
Yes	39 (49.4)	32 (56.1)	7 (31.8)	
GGT (U/L, median, IQR)	99 (75–155)	103 (77–158)	88.5 (72–133)	0.306
LSM (kPa, median, IQR)	6.3 (5.3–9.3)	6.2 (5.4–9.3)	7.15 (4.9–10.1)	0.922
CAP (dB/m, median, IQR)	265 (224–313)	261 (225–304)	288.5 (224–336)	0.223
ALB (g/dL, median, IQR)	4.3 (4.1–4.5)	4.3 (4.1–4.5)	4.3 (4.1–4.4)	0.908
AST (U/L, median, IQR)	32 (23–45)	32 (23–43)	31.5 (24–64)	0.835
ALT (U/L, median, IQR)	26 (19–38)	26 (20–34)	26 (19–41)	0.526
T‐Bil (mg/dL, median, IQR)	0.56 (0.46–0.74)	0.56 (0.44–0.75)	0.58 (0.48–0.74)	0.710
ALP (U/L, median, IQR)	64 (53–78)	62 (52–78)	67 (55–78)	0.460
Chole (mg/dL, median, IQR)	178 (152–208)	169 (150–205)	197 (171–219)	0.069
TG (mg/dL, median, IQR)	172 (114–296)	197 (125–344)	151 (104–269)	0.170
HDL (mg/dL, median, IQR)	48 (41–59)	48 (40–58)	48 (42–66)	0.447
LDL (mg/dL, median, IQR)	98 (71–126)	93 (65–116)	121 (90–136)	0.015
HBA1C (%, median, IQR)	5.7 (5.5–6.1)	5.7 (5.5–6)	5.95 (5.7–6.4)	0.018
PT (second, median, IQR)	10.5 (10–11)	10.45 (9.95–10.9)	10.6 (10.3–11)	0.264
AFP (ng/mL, median, IQR)	3.91 (2.7–5.89)	4.36 (2.78–6.14)	3.51 (2.7–4.91)	0.322
CDT (median, IQR)	4.57 (3.77–6.04)	4.98 (4.23–6.48)	3.725 (3.45–4.51)	< 0.001
%CDT (median, IQR)	1.67 (1.51–2.22)	1.78 (1.57–2.55)	1.605 (1.4–1.67)	0.012

Abbreviations: AFP, alpha‐fetoprotein; ALB, albumin; ALP, alkaline phosphatase; ALT, alanine aminotransferase; AST, aspartate aminotransferase; Bil, bilirubin; BMI, body mass index; CAP, controlled attenuation parameter; CDT, carbohydrate‐deficient transferrin; Chol, cholesterol; HBA1C, glycated hemoglobin; HDL, high density lipid; LDL, low density lipid; LSM, liver stiffness measurement; MS, metabolic syndrome; PT, prothrombin time; TG, triglyceride.

### 3.2 The Diagnostic Performances and Validities

**FIGURE 1:**
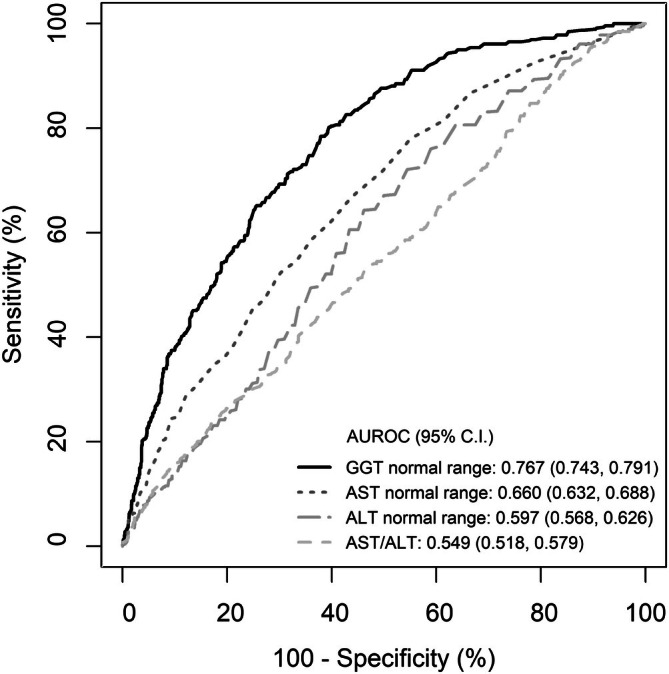
The performances of aspartate aminotransferase (AST), alanine aminotransferase (ALT), AST/ALT ratio, and gamma‐glutamyl transferase (GGT) in the detection of unhealthy alcohol use assessed with area under receiver operating characteristic curve (AUROC).

**FIGURE 2:**
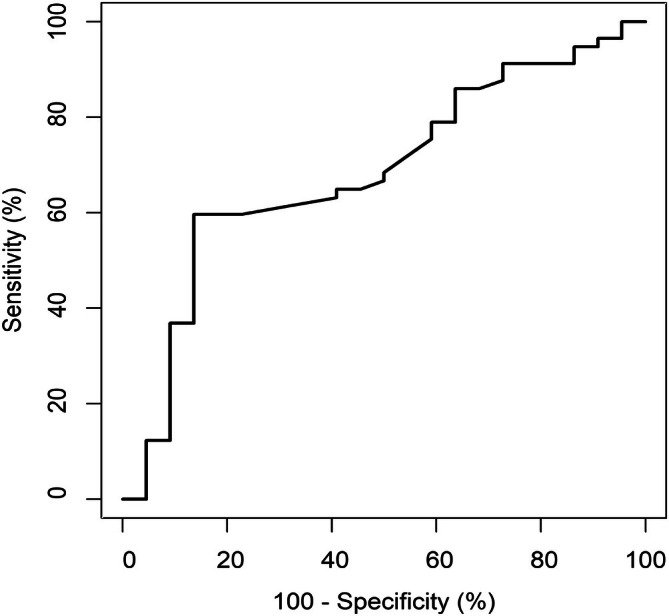
The performance of carbohydrate‐deficient transferrin percentage (%CDT) was 0.684 in the detection of unhealthy alcohol use assessed with area under receiver operating characteristic curve (AUROC).

With AUDIT score as reference, GGT, AST, ALT, and AST/ALT ratios in identification of UAU were 0.767, 0.660, 0.597, and 0.549, respectively, for the retrospective cohort of 1775 residents (Figure 1). For GGT in identifying UAU, there was higher performance for females compared to males (0.804 vs. 0.723). With the cutoff of GGT ≥ upper normal limit (UNL), the sensitivity, specificity, accuracy, positive diagnostic value (PPV), and negative diagnostic value (NPV) in the UAU detection was 62.5%, 75.5%, 72.1%, 47.4%, and 85.0%, respectively (Table 3). For the prospective cohort of 79 residents with sequential exam, %CDT in the identification of UAU was 0.684 (Figure 2). With the cutoff of 1.72, the sensitivity, specificity, accuracy, PPV, and NPV was 59.6%, 81.8%, 65.8%, 89.5%, and 43.9%, respectively (Table 4).

**TABLE 3:** The diagnostic validity with different cutoff for gamma‐glutamyl transferase in the detection of unhealthy alcohol use.

Cutoff	≥ 1 × UNL (95% CI)	≥ 2 × UNL (95% CI)	≥ 3 × UNL (95% CI)
Total (*n* = 1755)			
Sensitivity	0.625 (0.579, 0.670)	0.368 (0.324, 0.414)	0.240 (0.201, 0.281)
Specificity	0.755 (0.730, 0.778)	0.908 (0.891, 0.923)	0.946 (0.932, 0.958)
Accuracy	0.721 (0.699, 0.742)	0.767 (0.746, 0.787)	0.761 (0.741, 0.781)
PPV	0.474 (0.434, 0.515)	0.587 (0.528, 0.644)	0.611 (0.536, 0.683)
NPV	0.850 (0.828, 0.871)	0.802 (0.781, 0.822)	0.778 (0.757, 0.799)
Male (*n* = 792)			
Sensitivity	0.639 (0.570, 0.705)	0.365 (0.300, 0.435)	0.240 (0.184, 0.304)
Specificity	0.687 (0.647, 0.724)	0.868 (0.838, 0.895)	0.918 (0.893, 0.939)
Accuracy	0.674 (0.640, 0.707)	0.736 (0.704, 0.767)	0.740 (0.708, 0.770)
PPV	0.421 (0.366, 0.477)	0.497 (0.415, 0.579)	0.510 (0.407, 0.613)
NPV	0.842 (0.807, 0.874)	0.793 (0.760, 0.824)	0.772 (0.739, 0.803)
Female (*n* = 963)			
Sensitivity	0.614 (0.550, 0.674)	0.371 (0.311, 0.434)	0.239 (0.188, 0.297)
Specificity	0.810 (0.780, 0.839)	0.941 (0.921, 0.957)	0.969 (0.954, 0.981)
Accuracy	0.759 (0.731, 0.786)	0.792 (0.765, 0.818)	0.779 (0.751, 0.805)
PPV	0.533 (0.474, 0.592)	0.689 (0.604, 0.766)	0.732 (0.622, 0.824)
NPV	0.856 (0.827, 0.882)	0.809 (0.781, 0.835)	0.783 (0.754, 0.810)

Abbreviations: 95% CI, confidence interval; NPV, negative predictive value; PPV, positive predictive value; UNL, upper normal limit.

**TABLE 4:** The performance, cutoffs, and diagnostic validities of CDT% in the prediction of unhealthy alcohol use in residents with high gamma‐glutamyl transferase (*n* = 79).

Cutoff	Youden index	
	1.72 (95% CI)	2.5 (95% CI)
AUROC	0.684 (0.552, 0.816)	0.684 (0.552, 0.816)
Sensitivity	0.596 (0.458, 0.724)	0.263 (0.155, 0.397)
Specificity	0.818 (0.597, 0.948)	0.909 (0.708, 0.989)
Accuracy	0.658 (0.543, 0.761)	0.443 (0.331, 0.559)
PPV	0.895 (0.752, 0.971)	0.882 (0.636, 0.985)
NPV	0.439 (0.285, 0.603)	0.323 (0.209, 0.453)

Abbreviations: 95% CI, confidence interval; AUROC, area under receiver operating characteristic (ROC) curve; CDT%, carbohydrate‐deficient transferrin percentage; GGT, gamma‐glutamyl transferase; NPV, negative predictive value; PPV, positive predictive value; UAU, unhealthy alcohol use.

### 3.3 Liver Injury Related to High GGT

With ultrasonography, hepatic cirrhosis was diagnosed in 12 (15.2%) residents, including 10 (17.5%) with UAU and two (9.1%) without UAU. Advanced chronic liver disease was diagnosed in 19 (24.1%) residents by liver stiffness measurement. There was no significant difference between residents with and without UAU in the proportion of residents with hepatic cirrhosis by ultrasound and advanced chronic liver disease by liver stiffness measurements (Table 5). Two residents with UAU were referred to the medical center for the detection of liver nodules and cirrhosis at ultrasonography.

**TABLE 5:** Liver injury related to high gamma‐glutamyl transferase for patients with and without unhealthy alcohol use (UAU).

	Total	UAU	Non‐UAU	
	(*n* = 79)	(*n* = 57)	(*n* = 22)	
LS (kPa, %)				0.677
< 10	60 (75.9%)	44 (77.2%)	16 (72.7%)	
≥ 10	19 (24.1%)	13 (22.8%)	6 (27.3%)	
US LC (%)				0.493
No	67 (84.8%)	47 (82.5%)	20 (90.9%)	
Yes	12 (15.2%)	10 (17.5%)	2 (9.1%)	
CAP (dB/m, %)				
≥ 275	35 (44.3)	24 (42.1)	11 (50.0)	0.704
< 275	44 (55.7)	33 (57.9)	11 (50.0)	

Abbreviations: CAP, controlled attenuation parameter; LS, liver stiffness; US LC, ultrasound liver cirrhosis.

## 4 Discussion

Gamma‐glutamyl transferase is relatively expressed across tissues with the highest intracellular concentration in the liver, has been a part of the liver function tests, and is strongly associated with chronic alcohol use [12]. While the sensitivity of GGT was 37%–95%, the combination with %CDT might have increased the sensitivity in the detection of UAU [10, 13]. With AUDIT as a reference, this study demonstrated that high GGT was useful in the screening of UAU with a performance of 0.767 in the indigenous communities. Using the cutoff of GGT ≥ UNL, the NPV was 85% in the identification of UAU, and for sequential %CDT determination in high GGT, the PPV was 89.5% in identifying UAU. High GGT was related to UAU in 72.1%. Although more residents with UAU had cirrhosis by US, there was no significant difference in the severe hepatic fibrosis or cirrhosis diagnosis compared to those without UAU.

In Taiwan, a national survey showed that the population's prevalence of harmful alcohol use was 12.45% and 9.85% in 2014 and 2018, respectively [14], and although there were geographical variations, indigenous areas exhibited a high prevalence of 40%–55% [15, 16] through psychiatric interviews conducted more than 30 years ago. With a structured and validated ADUIT, our study showed that the prevalence of UAU was 7.4% and 44% in these two indigenous townships, where some indigenous areas had similar prevalence to that of non‐indigenous areas. While males had a much higher prevalence in UAU in a national survey [14], our study showed similar prevalence of UAU between males and females in these two indigenous communities. AUDIT is recommended as the screening tool for UAU detection in primary care, in which hazardous drinking is defined as AUDIT ≥ 8 for adults [2, 7]; however, women are more susceptible than men to alcohol‐induced injury [3], so in this study, UAU was defined as AUDIT ≥ 4 for females according to the AUDIT version recommended by the Taiwan Ministry of Health and Welfare [17]. While lower AUDIT in the detection of UAU for females might explain similar UAU between males and females in these two indigenous areas, the significant rise of harmful alcohol use for females reported in the national survey was another contributory factor [14]. This data might be important in policy‐making for alcohol prevention and intervention in indigenous communities.

Professional societies have recommended AUDIT as the screening tool of UAU with reported sensitivity and specificity between 70% and 85% [2, 7, 18]. However, alcohol intake might be underestimated due to fear of attached stigmata and recall bias at self‐reported measures or interviews, so alcohol biomarkers are necessarily supplementary measures in alcohol use surveys to detect UAU [8]. Commonly used laboratory tests, including aminotransferase, GGT, and %CDT, are simple and useful, but not ideal, to detect excessive alcohol use [9]. Although CDT and GGT had comparable performances, combined gamma‐CDT improved the sensitivity in the detection of excessive alcohol consumption levels compared with the single marker [10]. To obtain gamma‐CDT, mathematical calculation was necessary and might therefore limit its usefulness in community screening [10]. This study demonstrated that GGT had better performance than aminotransferase to screen UAU, which is similar to a previous review of alcohol biomarkers [8].

Taking resource limitations into consideration, sequential %CDT was performed for the prospectively enrolled patients with high GGT in our study. In clinical practice, the cutoff of GGT varies in different laboratories. The cutoff of %CDT is in a range of 1.7–2.5. While the optimal cutoff of GGT is expressed as the times of UNL, %CDT is determined by the Youden index assessed with ROC. In this study, the sensitivities were suboptimal with the proposed cutoffs, which might be affected by drinking patterns and metabolism variations. There might be no elevated GGT and %CDT in patients with binge drinking or chronic drinking with small amounts of alcohol. However, we demonstrated that the GGT value within the normal range had high value in excluding those without UAU, and sequential %CDT more than 1.72 for those with high GGT levels had high value in identifying harmful alcohol use. This study supported the use of biomarker‐guided, two‐step alcohol screening strategies in underserved or remote communities, especially where AUDIT questionnaires alone might be insufficient or underutilized. GGT might be used as a broad screening test, while %CDT offers diagnostic confirmation when needed. This proposed method might be feasible and affordable, and could be integrated with mobile health teams, ultrasound outreach, and brief interventions.

Although the indirect markers of GGT and %CDT are useful in UAU screening, direct alcohol biomarkers might have better performance [19]. A large general population study has demonstrated that blood phosphatidyl ethanol concentration, one of the direct alcohol biomarkers, increases with the alcohol unit consumed and the frequencies of alcohol consumption or binge drinking, and has high sensitivity and specificity in the detection of harmful alcohol consumption [20]. However, the utility of direct alcohol markers in community screening will need to be explored and validated.

In addition to being a marker of chronic alcohol ingestion and liver dysfunction, GGT is an indirect marker for cardiovascular and metabolic risk [12]. For patients with high GGT in this study, 72.2% and 49.4% revealed UAU and metabolic syndrome respectively. Instead of viral hepatitis with liver dysfunction, high GGT is commonly related to UAU and/or metabolic risk in these indigenous communities where viral hepatitis was endemic [11]. Although there are complex interactions, many studies have pointed to synergistic effects between harmful alcohol use and metabolic syndrome in liver‐related outcomes including cancer [21]. As a useful tool in screening UAU, GGT might have the additional effect of screening metabolic syndrome, which might increase the risk of liver‐related outcomes in these indigenous areas. Despite UAU patients revealing more patients with liver cirrhosis by ultrasound, there is no significant difference in the liver disease severity between UAU and non‐UAU cases assessed with transient elastography and ultrasonography. Further evaluation with more patients might be necessary to clarify this issue.

There were limitations in this study. One of the limitations was limited patient enrollment for sequential %CDT. A large cohort might be necessary to validate the use of sequential %CDT for patients with high GGT. While only GGT‐elevated individuals proceeded to %CDT testing in this study, it might introduce verification bias and limit generalizability. Although GGT and sequential %CDT were useful for UAU screening, the diagnostic validities will need to be validated in non‐indigenous areas where UAU prevalence might be lower than indigenous areas.

## 5 Conclusions

In summary, there is variation in UAU prevalence between indigenous areas in Taiwan using AUDIT as a reference. In contrast to the national survey, similar UAU prevalence between males and females in indigenous areas was found. Our findings suggest that GGT and sequential %CDT are useful for screening UAU in indigenous communities. GGT offers a high negative predictive value and is suitable for initial screening, while %CDT demonstrates a high positive predictive value for confirming UAU. This two‐step biomarker‐based approach may enhance early detection and intervention in resource‐limited, high‐risk populations.

## Ethics Statement

This study was approved by the Institutional Review Board of Kaohsiung Chang Gung Memorial Hospital and carried out in compliance with Helsinki declaration.

## Conflicts of Interest

The authors declare no conflicts of interest.

## Supporting information


**Data S1:** kjm270119‐sup‐0001‐Supinfo.docx.

## Data Availability

The data that support the findings of this study are available on request from the corresponding author. The data are not publicly available due to privacy or ethical restrictions.
