# Supplementary material for: Usefulness of Gamma‐Glutamyl Transferase and Sequential Carbohydrate‐Deficient Transferrin for Unhealthy Alcohol Use Screening in Indigenous Communities
Source: Kaohsiung J Med Sci. 2025 Oct 15;42(4):e70119. doi: 10.1002/kjm2.70119 (PMC13147956; doi:10.1002/kjm2.70119)
Supplement: Supplementary file 1 — Data S1: kjm270119‐sup‐0001‐Supinfo.docx. [file KJM2-42-e70119-s001.docx]

Supplement
